# Supplementary material for: Mechanistic Insights into Mancozeb-Induced Redox Imbalance and Structural Remodelling Affecting the Function of Human Red Blood Cells
Source: Antioxidants (Basel). 2025 Oct 23;14(11):1274. doi: 10.3390/antiox14111274 (PMC12649661; doi:10.3390/antiox14111274)
Supplement: Supplementary file 1 [file antioxidants-14-01274-s001.zip › antioxidants-3923745-supplementary.pdf]

SUPPLEMENTARY MATERIALS

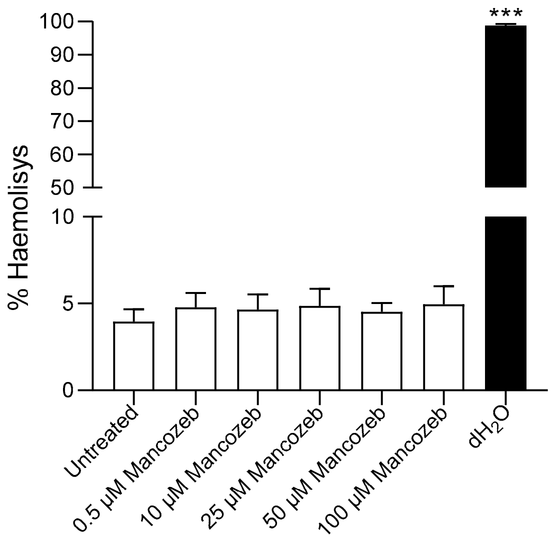

Figure S1. Detection of percentage of haemolysis in mancozeb-treated RBCs.

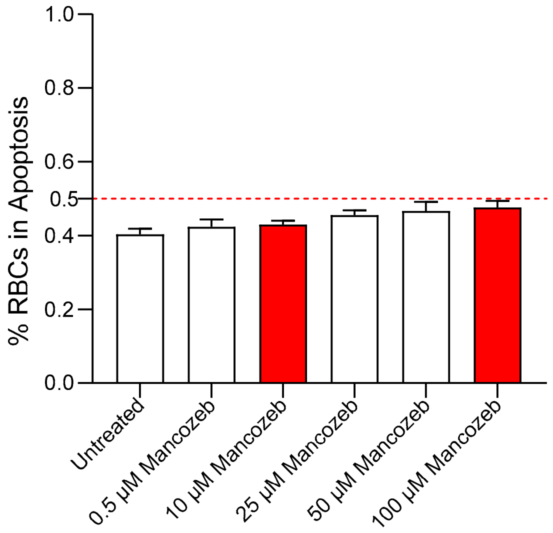

Figure S2. Detection of apoptosis by flow cytometry in mancozeb-treated RBCs.

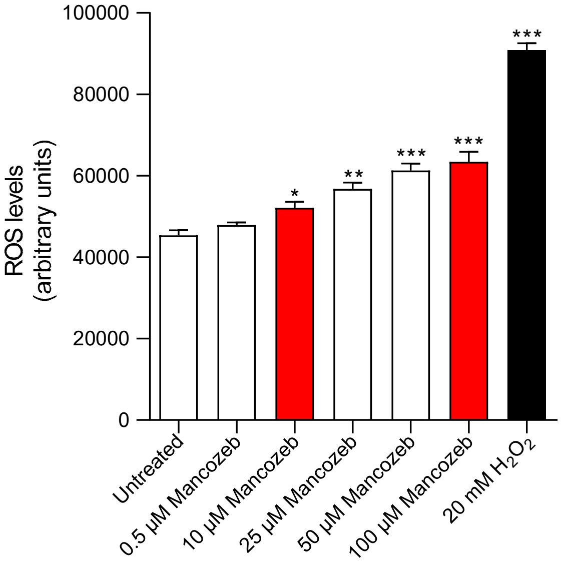

Figure S3. Detection of intracellular ROS levels in mancozeb-treated RBCs.
